# Elucidation of Motifs in Ribosomal Protein S9 That Mediate Its Nucleolar Localization and Binding to NPM1/Nucleophosmin

**DOI:** 10.1371/journal.pone.0052476

**Published:** 2012-12-20

**Authors:** Mikael S. Lindström

**Affiliations:** Department of Oncology-Pathology, Karolinska Institutet, Stockholm, Sweden; Université Libre de Bruxelles. BELGIQUE, Belgium

## Abstract

Biogenesis of eukaryotic ribosomes occurs mainly in a specific subnuclear compartment, the nucleolus, and involves the coordinated assembly of ribosomal RNA and ribosomal proteins. Identification of amino acid sequences mediating nucleolar localization of ribosomal proteins may provide important clues to understand the early steps in ribosome biogenesis. Human ribosomal protein S9 (RPS9), known in prokaryotes as RPS4, plays a critical role in ribosome biogenesis and directly binds to ribosomal RNA. RPS9 is targeted to the nucleolus but the regions in the protein that determine its localization remains unknown. Cellular expression of RPS9 deletion mutants revealed that it has three regions capable of driving nuclear localization of a fused enhanced green fluorescent protein (EGFP). The first region was mapped to the RPS9 N-terminus while the second one was located in the proteins C-terminus. The central and third region in RPS9 also behaved as a strong nucleolar localization signal and was hence sufficient to cause accumulation of EGFP in the nucleolus. RPS9 was previously shown to interact with the abundant nucleolar chaperone NPM1 (nucleophosmin). Evaluating different RPS9 fragments for their ability to bind NPM1 indicated that there are two binding sites for NPM1 on RPS9. Enforced expression of NPM1 resulted in nucleolar accumulation of a predominantly nucleoplasmic RPS9 mutant. Moreover, it was found that expression of a subset of RPS9 deletion mutants resulted in altered nucleolar morphology as evidenced by changes in the localization patterns of NPM1, fibrillarin and the silver stained nucleolar organizer regions. In conclusion, RPS9 has three regions that each are competent for nuclear localization, but only the central region acted as a potent nucleolar localization signal. Interestingly, the RPS9 nucleolar localization signal is residing in a highly conserved domain corresponding to a ribosomal RNA binding site.

## Introduction

The nucleolus is a distinct subnuclear compartment first described a few hundred years ago [1], [2]. Nucleoli form around tandem repeated ribosomal DNA genes from which the 47S ribosomal RNA (rRNA) precursor is transcribed. The 47S rRNA is processed into the 28S, 18S and 5.8S rRNA. These rRNAs assemble with the 5S rRNA and ribosomal proteins (RPs) into pre-ribosomes with the help of an array of non-ribosomal proteins [3]. While the 47S rRNA is made in the nucleolus, the RPs are synthesized by pre-existing ribosomes in the cytoplasm and therefore have to be imported into the nucleus. In general, proteins enter into the nucleus through the nuclear pore complex by specific interactions with import receptors whereas small proteins can move more freely through the pore complex [4]. Transport of proteins into the nucleus is typically mediated by one or several nuclear localization signals (NLS). A classic NLS contains a cluster of basic amino acids, usually lysine or arginine, organized in either a single stretch (the mono-partite NLS) or in two stretches (the bi-partite NLS), where two small clusters are separated by a few amino acids. The mechanisms of nuclear import of RPs are not completely understood, but it is an active process assisted by a range of import factors, rather than occurring by passive diffusion [5], [6]. Once the RPs have entered the nucleus they localize to the nucleolus. RPs are not the only proteins to be found in the nucleolus. High resolution mass spectrometry revealed that the nuclear genome of human cells encodes for around 4,500 proteins with potential for nucleolar localization [7], [8]. Analysis of the nucleolar proteome revealed functionally diverse proteins previously implicated in ribosome biogenesis, but also in the regulation of other cellular processes including chromatin remodeling, export of mRNA, assembly of various ribonucleoprotein complexes, cell cycle control, and protein turnover [2], [3], [7], [9]–[11]. In addition, quantitative proteomics revealed that the nucleolar proteome is not static but changes upon different growth conditions or cellular stress [9]. For instance, a live cell imaging study of RPs fused to GFP showed that they are in a state of dynamic exchange, rapidly shuttling between the nucleolus and nucleoplasm [12]. The exchange of proteins and RNA is presumably facilitated by the lack of a classical lipid bi-layer membrane around the nucleolus. RPs are abundantly expressed and can be incorporated into ribosomal subunits, or subjected to rapid degradation if not needed, for instance when the synthesis of rRNA is inhibited [9], [12].

While the nuclear import of proteins usually relies on one or several NLS, a consensus motif for nucleolar targeting has not been found [11], [13], [14]. As a matter of fact, a specific mechanism for targeting of proteins into the nucleolus may not be required because the nucleolus lacks a lipid membrane. Nevertheless, nucleolar localization is often mediated by amino acid motifs with a high content of repeated basic residues. Motifs for nucleolar localization can also be more complex. For example, the nucleolar localization of a protein can be affected by mutations in residues critical to maintain a proteins normal folding [15]–[17], by changes in pH/oxygen levels [18], [19], and by various post-translational modifications for example phosphorylation [20] and neddylation [21]. Binding to other proteins may expose cryptic nucleolar localization signals that are hidden [22]. Taken together, nucleolar localization of a protein is often a result of a complex series of interactions and modifications.

The amino acid sequence motifs required for nucleolar localization are known in the literature as nucleolar targeting signals, nucleolar binding domains, nucleolar retention motifs, or nucleolar localization signals (NoLS). The choice of term depends upon how one sees the nucleolar assembly mechanism. As mentioned, nucleolar proteins including RPs can associate with, and dissociate from, nucleolar components in a dynamic exchange with the surrounding nucleoplasm [12], [23]. Hence, nucleolus-specific retention mechanisms for proteins exist and the residence time of a protein in the nucleolus is likely to rely on its specific interactions [24]. As such, the NoLS can be regarded as a nucleolar molecule-interacting sequence rather than a specific targeting signal [25]. NoLS have been found in many nucleolar proteins for example the tumor suppressor p14ARF [26], viral proteins [27], and in a subset of RPs [28]–[31]. Part of the quest to understand the dynamics of nucleolar proteins involves mapping and characterization of NoLS. Although NoLS are divergent in sequence, recent studies show that it is to some extent possible to predict whether a protein may be destined for the nucleolus because these motifs do share certain features [13], [14]. So what is the function of the NoLS? Well, the NoLS may mediate binding to rRNA, rDNA, or other proteins in the nucleolus through electrostatic interactions, in line with the nucleolar retention model for nucleolar assembly. For example, some of the RPs are likely to be retained within the nucleolus by binding to the rRNA transcript [12]. Similarly, some transiently nucleolar proteins may be detained within the nucleolus by binding to noncoding RNA produced by the nucleolar intergenic spacer [32].

A second model for how NoLSs may function exists. A class of acidic nucleolar proteins can shuttle between the nucleus and cytoplasm [33]. These acidic proteins may actively recruit NoLS containing proteins from their site of synthesis in the cytoplasm and escort them to the nucleolus [34]–[36] [37], a function in support of the targeting model for nucleolar localization. NPMI (also known as B23, NO38 or nucleophosmin) is one of these acidic nucleolar proteins, and it is an abundant phosphoprotein localized mainly in the nucleoli during interphase. NPM1 is a multifunctional protein with nucleic acid binding, ribonuclease and molecular chaperone activities [38] and that can interact with many other nucleolar proteins [39]. NPM1 was hypothesized to play a role in the cellular transport of NoLS containing protein [33], [40], [41].

Ribosomal protein S9 (RPS9, GI:14141193), RPS4 in prokaryotes (GI:323376825), was previously shown to be a novel NPM1 interacting protein [42]. RPS9 is one of the first proteins that directly bind to the 18S rRNA [43], [44]. Structural studies of the ribosomes in prokaryotes and eukaryotes (yeast) revealed that RPS4/RPS9 is located at the head of the small ribosomal subunit near the entrance tunnel of the mRNA leading into the decoding center, and being in close contact with rRNA [45]–[47]. However, there are structural features in the entrance tunnel that are unique to eukaryotes, for example the orientation of RPS9 is different [45]. RPS9 has been implicated translation elongation steps including mRNA unwinding and decoding accuracy, and prokaryotic ribosomes with mutations in RPS4 possess a lower ability to unwind RNA duplexes [48]–[50]. While the function of RPS9 in mature ribosome function might be related to translational fidelity, not only until recently, did the function of RPS9 in yeast and mammalian cell ribosome biogenesis become better understood. RPS9 is required in the early steps of ribosome biogenesis and knockdown of RPS9 results in the accumulation of the 45S and 30S pre-rRNAs, indicating the failure of all the processing steps in the 5′-external transcribed spacer and in the internal transcribed spacer region 1 (ITS1) necessary to generate 18S rRNA beyond cleavage of ITS1 at site 2 [43], [51], [52]. Depletion of RPS9 by small interfering RNA in human cancer cell lines resulted in decreased global protein synthesis in association with induction of p53 target genes followed by cell cycle arrest or apoptosis depending on cell type [53].

RPS9 can be detected in the nucleoli and cytoplasm of mammalian cells [42], [54], [55]. This dual site pattern is often observed with other RPs and is thought to correspond to ribosome biogenesis in the nucleolus and to RPs that have been incorporated within mature cytoplasmic ribosomes [55], [56]. At present there is an increasing interest in the process of ribosome biogenesis and the functions of ribosomal proteins. To determine the motifs that mediate the cellular localization of RPS9 may provide additional clues to a better understanding of its role in ribosome biogenesis and translation.

## Materials and Methods

### Cell Line, Plasmids and Transfection Procedures

U2OS osteosarcoma cells were obtained from American Type Culture Collection (ATCC) and cultured in Iscovés medium supplemented with 10% fetal bovine serum, 2 mM L-glutamine and penicillin-streptomycin in a +37°C humidified incubator. RPS9-FLAG3C, EGFP (C1)-RPS9, RPS9-EGFP (N1), and RPS9-FLAG3C deletion and point mutants were created by PCR and site directed mutagenesis PCR. NPM1 (B23) expression plasmids (GST-NPM1, Myc-tagged wt NPM1 and ΔNLS-NPM1) were described previously [15], [42] or created by PCR. Primer sequences, PCR conditions, and plasmid maps are available upon request. Plasmids were transfected into cells by using Lipofectamine2000 reagent according to the manufacturer (Life Technologies).

### Immunofluorescence Staining

For immunofluorescence studies, cells were seeded on coverslips in 6-well plates and transfected. At 24 hours after transfection, the cells were washed in PBS and fixed in formalin solution containing 4% w/v formaldehyde (Sigma-Aldrich), permeabilized with 0.2% Triton X-100 for three minutes, and kept for 30 minutes in blocking buffer (0.5% bovine serum albumin in 1× PBS). Next, the cells were incubated 60 minutes with primary antibodies in blocking buffer followed by three washes in PBS and a 30 minutes exposure to FITC- or Texas Red-conjugated anti-mouse or anti-rabbit secondary IgG (Vector). After three PBS washes and one final wash in distilled water, the coverslips were mounted onto glass slides with Vectashield anti-fading agent (Vector) containing DAPI.

### Microscopy

Immunostained cells were analyzed by using a Zeiss Axioplan II microscope equipped with Plan-Apochromat 63×/1.4 and Plan-Neofluar 100x/1.30 objectives, under the control by Axiovision 3.1 software. An Olympus microscope equipped with a Leica DFC320 camera and a PlanApo 40 objective (Olympus) was dedicated to the analysis and documentation of AgNOR and Toluidine Blue stained cells. For live cell imaging, an inverted Nikon Eclipse TS100 (S Plan Fluor 30x/0.60 and LWD 20x/0.40 objectives) with an Infinity camera was used. Images were assembled in Adobe Photoshop CS5.

### Antibodies

The following antibodies were used: mouse anti-Myc (clone 9E10, Sigma-Aldrich), rabbit anti-Myc 9E10 epitope (Abcam), mouse anti-FLAG (clone M2, Sigma-Aldrich), rabbit anti-FLAG (Sigma-Aldrich), mouse anti-β-actin (clone AC15, Sigma-Aldrich), mouse anti-BrdU (clone BU33, Sigma-Aldrich), mouse anti-NPM1 (Invitrogen), rabbit anti-fibrillarin (Abcam), mouse anti-GFP (Clontech), and rabbit anti-RPS9 (S9-162) [42].

### Chemicals

Transcription inhibitor Actinomycin D was prepared as a 1 mM stock solution in DMSO and used at a final concentration of 5 nM. Proteasome inhibitor MG132 was dissolved in ethanol and used at 10 µM final concentration. All compounds were purchased from Sigma-Aldrich.

### Ag-NOR and Acidic Toluidine Blue O Staining

For silver staining of nucleolar organizer regions (Ag-NOR staining), cells grown on coverslips were fixed in 2% glutaraldehyde in PBS for 10 minutes at room temperature, rinsed with PBS and once in distilled water, followed by fixation for 5 minutes with methanol:acetic acid 3∶1, again rinsed in distilled water and subsequently stained with a mix of 1 part A and 2 parts B for 45 minutes in the dark at room temperature (solution A: 2% gelatin in 1% formic acid and solution B: 50% silver nitrate in ultrapure distilled water) as described [57]. The reactions were terminated after 40 minutes by washing in distilled water and the coverslips with cells mounted upside down on slides. Toluidine Blue O staining to detect nucleoli was carried out as described [57].

### BrdU Incorporation Assay

For estimation of DNA replication activity, U2OS cells were seeded at an equal sub-confluent cell density in 6-well plates. Cells were transfected with wt or mutant RPS9-FLAG plasmids and after 18 hours bromodeoxyuridine (BrdU) (Sigma–Aldrich) was added (10 µg/ml) to the culture medium and cells were incubated in it for an additional 24 hours. Cells were fixed in methanol–acetone (1∶1 vol/vol), stained for FLAG using a rabbit polyclonal antibody, and then a secondary FITC anti-rabbit antibody. Stained cells were then treated for 15 min with 1.5 M HCl and extensively washed. Next, cells were stained for one hour with a mouse monoclonal anti-BrdU antibody, followed by a Texas Red-conjugated anti-mouse antibody.

### Western Blotting

Cells were harvested in Nonidet P-40 lysis buffer (50 mM Tris-HCl, pH 7.5, 150 mM NaCl, 0.5% Nonidet P-40, 50 mM NaF, 1 mM NaVO_3_, 1 mM dithiothreitol, 1x protease inhibitor cocktail (Sigma-Aldrich), 1 mM phenylmethylsulfonyl fluoride-PMSF). Samples were prepared by mixing with SDS-sample buffer and loaded on 10 or 12% Bis-Tris gels (Life Technologies). Proteins were blotted onto PVDF membranes, incubated in blocking solution with primary antibodies overnight followed by three washes in PBS. Membranes were subsequently incubated with secondary horse radish peroxidase conjugated antibody at room temperature for two hours and after three additional washes in PBS, proteins were visualized by using ECL reagent (Amersham Biosciences) or West Pico (Pierce).

### *In vitro* Protein-protein Interaction Assay

The coupled *in vitro* transcription and translation reactions were performed using the TnT kit as per the manufacturer's instructions (Promega). For *in vitro* binding assays individually translated proteins were mixed with GST-NPM1 expressed by the plasmid pGEX3X-NPM1 in BL21 bacteria. Expression and purification of GST fusion proteins were done according to the manufacturer's instructions (Amersham Biosciences). GST fusion proteins bound to glutathione-Sepharose 4B beads (Amersham Biosciences) were incubated and rotated overnight at +4°C with *in vitro* translated protein in binding buffer (0.2% Nonidet P-40, 150 mM NaCl, 1 mM PMSF). Following three extensive washes with rotation and shaking in binding buffer the beads were boiled and re-suspended in SDS-sample buffer, and the proteins resolved by SDS-PAGE, followed by staining of the gels with Coomassie Brilliant Blue, drying, and autoradiography.

### Bioinformatics

NoD, nucleolar localization sequence detector, provides predictions of nucleolar localization sequences in eukaryotic proteins (http://www.compbio.dundee.ac.uk/nod). Nuclear localization signals were predicted using PSORT II (http://www.psort.org/). NetNES 1.1 predicts leucine-rich nuclear export signals (NES) in eukaryotic proteins (http://www.cbs.dtu.dk/services/NetNES/). Multiple sequence alignments were made with ClustalW (http://www.ebi.ac.uk/Tools/msa/clustalw2/).

## Results

### RPS9 can be Detected in the Cytoplasm and Nucleoli

**Figure 1:**
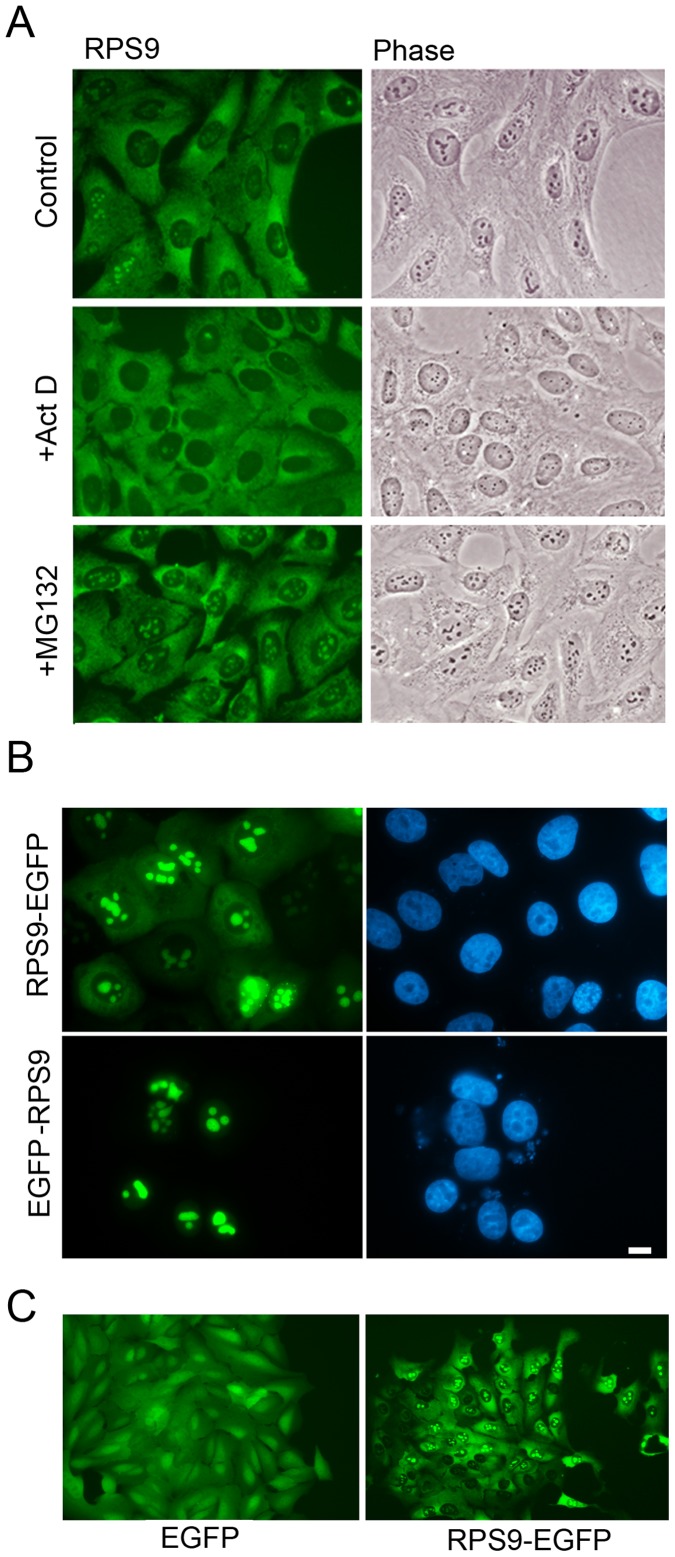
Cellular localization of endogenous RPS9 and RPS9-EGFP fusion proteins. A.) U2OS osteosarcoma cells were stained for endogenous RPS9 under normal cell culture conditions (upper panel), in cell cultures treated with a low concentration of Actinomycin D (5 nM) that selectively blocks RNA polymerase I activity (middle panel), and in cultures treated with proteasome inhibitor MG132 (lower panel). Objective 63x. B.) Plasmids encoding for EGFP-RPS9 or RPS9-EGFP was transfected into U2OS cells, fixed and counterstained with DAPI after 24 hours. Bar 10 µM. C.) U2OS cells were selected with G418 for 2 weeks to achieve uniform expression of EGFP or RPS9-EGFP, respectively. Objective 40x.

The localization of endogenous and ectopic RPS9 was analyzed in the U2OS osteosarcoma cell line. This line was chosen because of the adherent morphology of the cells with well-defined nuclear and nucleolar areas. The cells were stained with an antibody specific for human RPS9 which revealed a predominant cytoplasmic staining with a weaker dotty pattern in the nucleus (Figure 1A), in agreement with published data [42]. The dots correspond to nucleoli as judged by phase contrast microscopy images (Figure 1A). Cells were treated with Actinomycin D at a low concentration of 5 nM. This is a concentration that preferentially blocks RNA pol I transcription in nucleoli [58]. The treatment resulted in the nucleolar shrinkage and diminished nucleolar RPS9 staining (Figure 1A). Exposure of cells to proteasome inhibitor causes accumulation of ribosomal proteins in the nucleolus [9]. In agreement, the RPS9 nucleolar signal became more intense in cells treated with the proteasome inhibitor MG132 (Figure 1A).

RPS9 was fused with EGFP at its C-terminus (RPS9-EGFP) and the localization of the fusion protein was determined. RPS9-EGFP was predominantly nucleolar with a weaker but distinct cytoplasmic staining, excluding the nucleoplasm (Figure 1B). This was in contrast to the rather even intracellular EGFP (only) fluorescence seen (Figure 1C). RPS9 was also tagged with EGFP at its N-terminus (EGFP-RPS9). Although both EGFP-RPS9 and RPS9-EGFP localized efficiently to the nucleolus, it was found that EGFP-RPS9 presented with a much weaker cytoplasmic staining than RPS9-EGFP (Figure 1B). RPS9-EGFP therefore had a localization pattern that best resembled the endogenous RPS9 protein. The lack of cytoplasmic staining can be interpreted as a failure of the EGFP fusion protein to be incorporated into ribosomes. There are several other examples of ribosomal proteins fused at their N-termini with bulky tags, such as GFP or β-galactosidase, displaying localization patterns that do not mimic the the corresponding endogenous ribosomal protein [28], [55]. In contrast, fusions of ribosomal proteins with GFP at their C-termini often results in functionality and normal localization pattern [12], [59]–[61].

### Expression and Localization of RPS9-EGFP Fusion Proteins in Fixed and Living Cells

**Figure 2:**
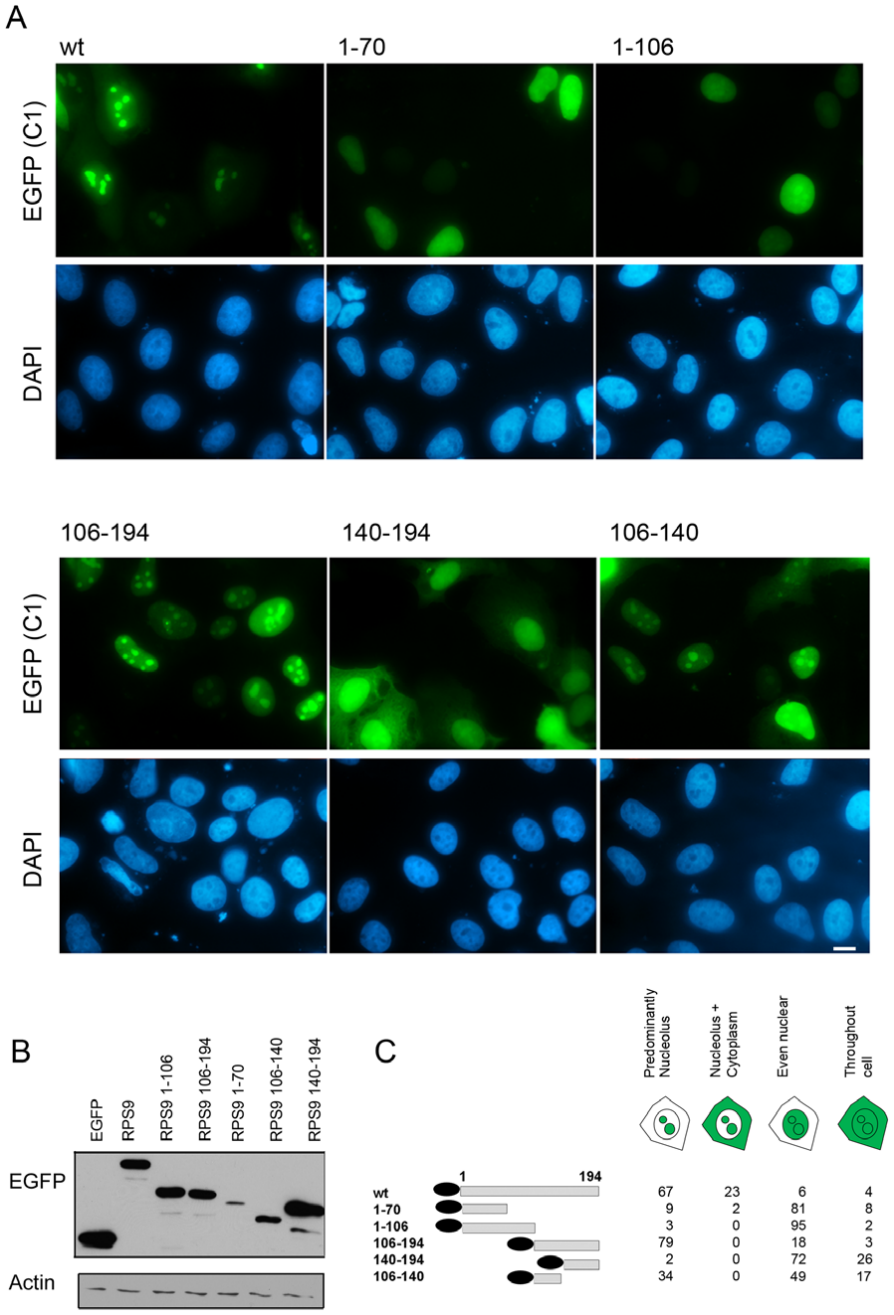
Expression and localization of EGFP-RPS9 fusion proteins. A.) U2OS osteosarcoma cells were transfected with EGFP-RPS9 deletion mutants as indicated in the figure and fixed 24 hours after transfection. Corresponding DAPI images are also shown. Bar 10 µM. B.) Western blotting demonstrates expression levels of the different EGFP-RPS9 fusion proteins. EGFP-RPS9 was detected using anti-GFP antibody and loading was verified with a β-actin antibody. C.) Schematic representation of the different fusion proteins tested and their localization pattern within cells. The patterns were defined as being predominantly nucleolar, nucleolar and cytoplasmic, even nuclear, or even throughout the entire cell. Shown is the percentage of cells with a particular staining pattern, representing the analysis of 200 transfected cells for each transfected plasmid.

**Figure 3:**
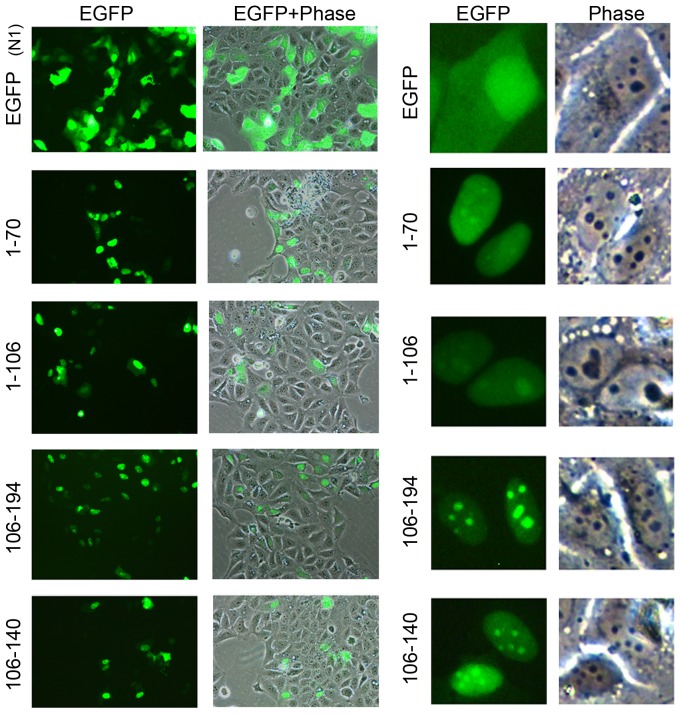
Expression and localization of RPS9-EGFP fusion proteins in living U2OS cells. U2OS cells were transfected with EGFP or EGFP-RPS9 deletion mutants as indicated in the figure and monitored under the inverted microscope 24 hours after transfection. Corresponding phase contrast images are shown and zoom-in of selected representative cells are in addition displayed on the right side. Objective 20x.

It is remarkable that ribosomal proteins display such a low variation in the proportion of basic residues and RPS9 is no different. It is composed of 194 amino acids and similar to other RPs it has a high content of basic amino acid residues 23.7% (Table S1). By the use of PSORT II to define putative NLSs in RPS9 two overlapping bi-partite and a mono-partite NLS were predicted to be present in RPS9. These motifs are the overlapping dual bipartite motif “KREVWRVKFTLAKIRKA“ at residue 40 and “RKAARELLTLDEKDPRR” at residue 54, denoted as NLS-1 and the mono-partite “PGRVKRK” at residue 170 (NLS-2) (Figure S1). It was of interest to compare RPS9 with all other small subunit RPs using PSORT II. Interestingly, 12 out of 31 RPs from the small subunit were not predicted to have any classical NLS (Table S1). Nevertheless, many of the apparently NLS-deficient RPs do localize to the nucleus and nucleolus suggesting a different design and complexity of their NLS. In order to experimentally define the sequences that mediate RPS9 nucleolar localization subcellular localization studies on wild type (wt) and mutant RPS9 proteins fused to EGFP were conducted. Expression plasmids that contain various RPS9 sequences fused in frame with EGFP (C1) had been made, with the thought that this would result in comparable expression levels because of an identical translation initiation sequence. The plasmids were transfected into U2OS cells and monitored for EGFP expression 24 hours post-transfection (Figure 2A). The various localizations of EGFP-RPS9 mutants are summarized in Figure 2C, and the expression levels of the different mutants were confirmed by Western blotting (Figure 2B). Predominantly nucleoplasmic staining, not excluding the nucleoli, was seen in cells expressing EGFP-RPS9^1–70^ (81% of transfected cells) and also in cells expressing EGFP-RPS9^1–106^ (95%) (Figure 2C). EGFP-RPS9^106–194^ and EGFP-RPS9^106–140^ localized efficiently to the nucleus and both fusion proteins clearly accumulated within nucleoli. EGFP-RPS9^140–194^ displayed a more diffuse nuclear staining. The localization pattern of EGFP-RPS9^1–106^ and EGFP-RPS9^140–194^ fusion proteins indicated that neither domain was sufficient for exclusive nucleolar targeting. Because of the bias in localization towards the nucleolus seen with EGFP (C1)-RPS9, a panel of RPS9-EGFP (N1) deletion constructs was made. The localization was determined in living non-fixed U2OS cells (Figure 3). Expression of EGFP alone resulted in an even cytoplasmic and nuclear signal although the intensity in the nucleus was slightly higher. Inspection of cells expressing the various RPS9-EGFP mutants again showed that a central region in RPS9 (aa 106–140) mediate nucleolar localization of EGFP. Also, the RPS9 fragment 1–70 as well as 1–106 can drive nuclear localization of EGFP but without nucleolar accumulation. Hence the difference in localization of RPS9 fused with EGFP at the N or C-terminus appears to apply to the full length protein only.

### A Central Domain is Required for RPS9 Nucleolar Accumulation

**Figure 4:**
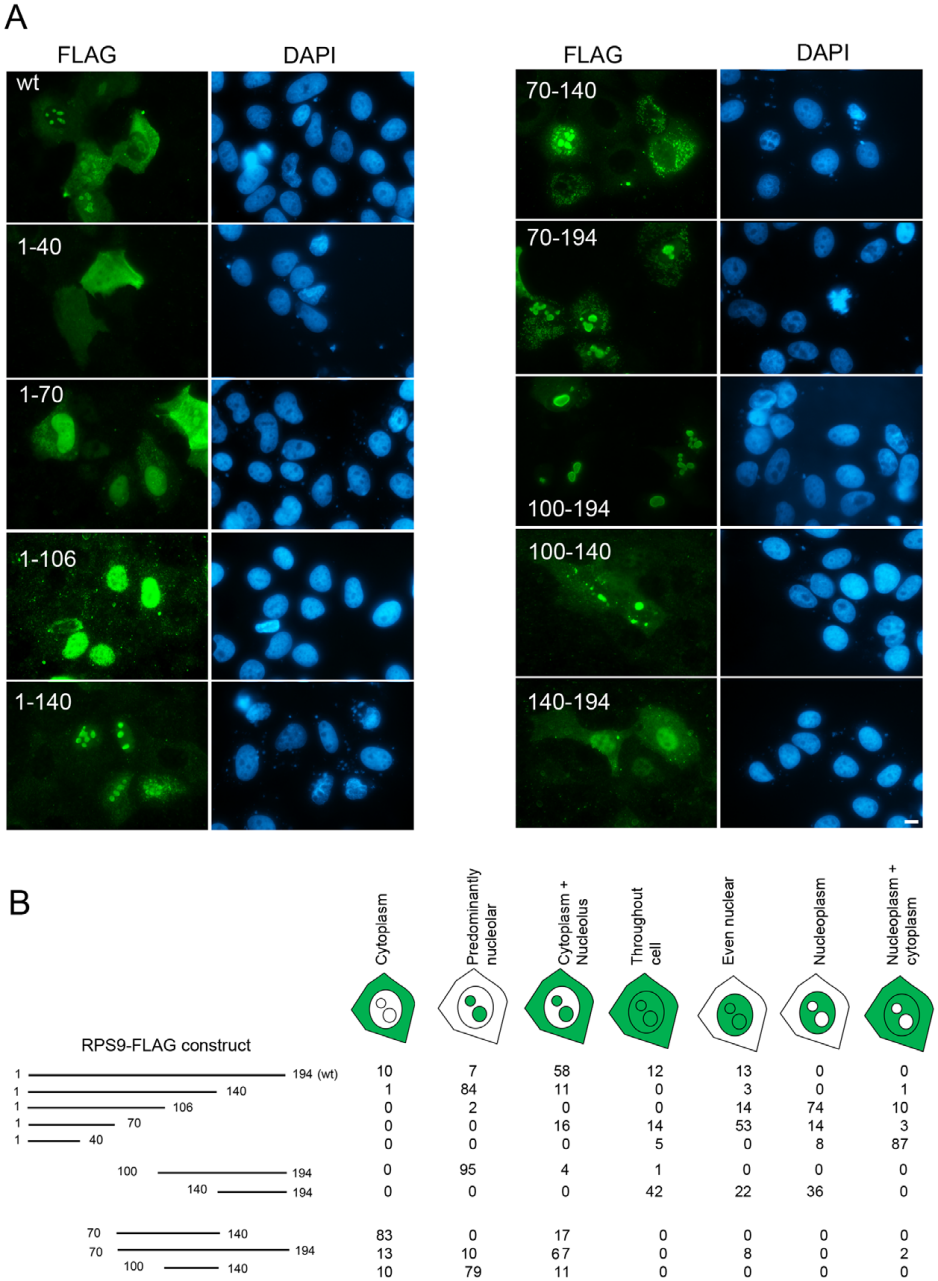
Localization of RPS9-FLAG and RPS9-FLAG deletion mutants. A.) U2OS osteosarcoma cells were transfected with wt RPS9-FLAG and RPS9-FLAG deletion mutants as indicated in the figure and fixed 24 hours after transfection. Corresponding DAPI images are shown. Bar 10 µM. B.) Schematic representation of the different RPS9-FLAG chimeric proteins tested and their respective localization pattern. The patterns were defined as being cytoplasmic, predominantly nucleolar, nucleolar and cytoplasmic, even nuclear, throughout the entire cell, nucleoplasmic excluding nucleoli, or nucleoplasmic and cytoplasmic but excluding the nucleoli. Shown is the percentage of cells with a particular pattern based on 200 transfected cells analyzed for each construct.

The results obtained with EGFP fusions warranted further investigation of NLS and NoLS domains in RPS9. That EGFP was not strictly excluded from the nucleolus was a caveat. To resolve this issue, RPS9 was tagged with Myc and FLAG epitopes, respectively. The background nuclear fluorescence signal from the FLAG (M2) antibody was less than when using the Myc antibody. In addition, RPS9 protein ends with an acidic amino acid patch (DDEEED). It was thought that the FLAG tag may work in harmony with this patch because the FLAG tag is acidic itself (DYKDDDDK). Because of this and the low FLAG antibody background, expression plasmids were constructed containing various RPS9 sequences fused in frame with a C-terminal 3x FLAG-tag. The RPS9-FLAG constructs were transfected into U2OS cells and monitored for expression 24 hours post-transfection by immunofluorescence staining (Figure 4). Localization of all of the FLAG constructs used is summarized in Figure 4B. Full length RPS9-FLAG localized to nucleoli and cytoplasm excluding the nucleoplasm. This staining pattern as seen in most of the transfected cells (58%) resembled that of endogenous RPS9 (Figure 1A), but other cellular staining patterns could be seen (Figure 4B). For instance, a minor fraction (13%) of the transfected cells displayed a rather even nuclear staining. This is probably an overexpression phenomenon because this localization pattern has not been observed with endogenous RPS9 or in stable cell lines expressing RPS9-EGFP (Figure 1). Overall, wt RPS9-FLAG, when transiently expressed, displayed the greatest heterogeneity in the staining patterns when compared with the mutants. The deletion mutants RPS9^1–70^-FLAG and RPS9^1–106^-FLAG were localized predominantly throughout the nucleoplasm, but it should be emphasizes that the RPS9^1–70^-FLAG protein was not excluded from the nucleolus and in fact could be detected at the nucleolar periphery in quite a few of the transfected cells. RPS9^140–194^-FLAG mutant was predominantly nucleoplasmic while RPS9^1–40^-FLAG was evenly distributed throughout the cells in majority of the transfected cells (87%), being excluded from the nucleoli. An intense nucleolar staining was seen in cells expressing RPS9^100–194^-FLAG, RPS9^1–140^-FLAG and RPS9^100–140^-FLAG, suggesting that a potent NoLS is to be found within the central domain of RPS9, which would be in agreement with the data obtained with RPS9-EGFP fusion proteins. Intriguingly, RPS9^70–140^-FLAG displayed a peculiar fine dotty cytoplasmic staining (Figure 4) that corresponded to mitochondria (Figure S3). A similar staining pattern was seen with RPS9^70–194^-FLAG (Figure 4). Both of the fusion proteins could be detected in nucleoli although much less frequently in the case of RPS9^70–140^. In search for an explanation of this cytoplasmic pattern, it was noted that the region 60–105 in RPS9 is hydrophobic and rich in leucine residues (Figure S1). In addition, this domain scores high in a computer based search for potential nuclear export signals (Figure S2).

That RPS9^100–194^-FLAG (but not RPS9^140–194^-FLAG), and EGFP-RPS9^106–140^ (but not EGFP-RPS9^1–106^ or EGFP-RPS9^140–194^) were targeted to nucleoli suggested that a central motif within aa 106–140 is important for the nucleolar localization of RPS9. This motif “LERRLQTQVFKLGLAKSIHHARVLIRQRHIRVRKQ” contains several conserved basic residues. Predicting NoLS in proteins is even more difficult but based on experimental data sets and computer programming advances have been made. One program that can be used to predict NoLS is the NoD program [62], [63]. In retrospect NoD was used to search for putative NoLSs in RPS9. The NoD predicts one region in RPS9 that barely reaches above the standard threshold namely “YGGGRPGRVKRKNAKKGQGG” between residues 165 and 184 (Figure S2). However, EGFP fused with this region did not accumulate in nucleoli. The NoLS containing central region was by NoD indicated to have an increased likelihood of nucleolar localization but the region did not reach above the set threshold (Figure S2). Next, all of the small subunit RPs were evaluated by NoD (Table S1) and it turned out that many RPs are not predicted to have a NoLS (19 out of 31 were negative) despite experimental evidence for their nucleolar presence [7]. However, NoD accurately predicted the correct NoLSs in RPS6 and RPS7 in line with experimental data published [64], [65].

**Figure 5:**
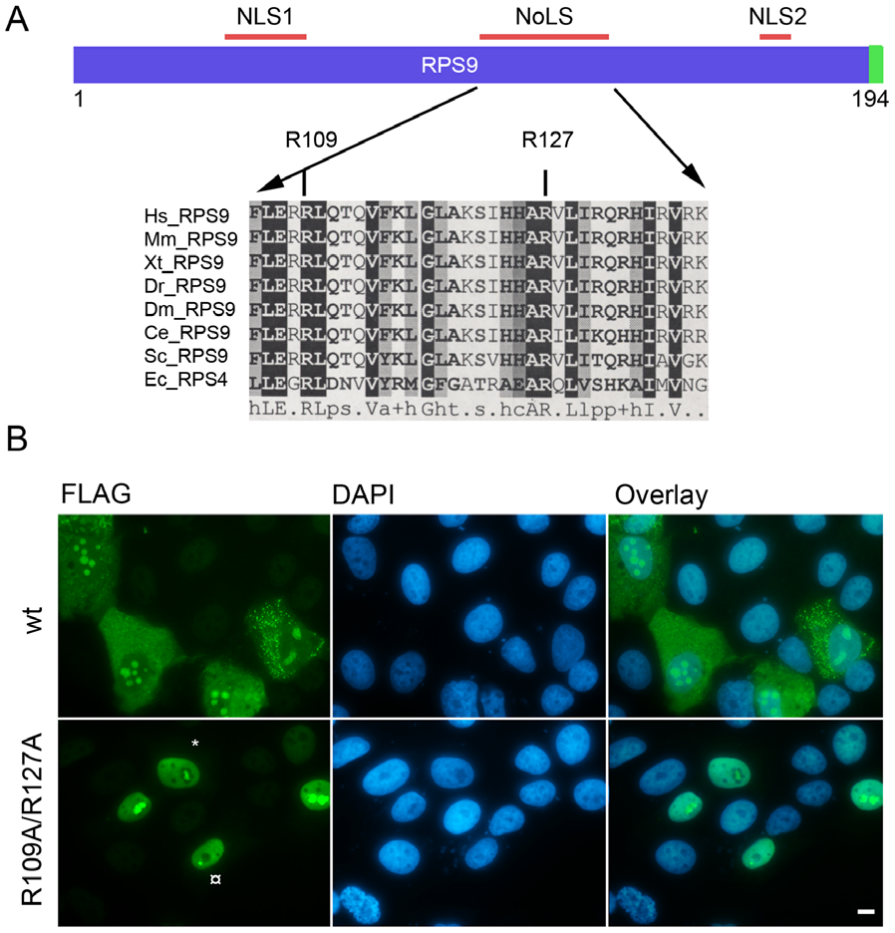
Altered localization of RPS9-FLAG NoLS domain mutant. A.) Schematic representation of RPS9 with the NLS1, NLS2 and NoLS. Below is a partial multiple sequence alignment of RPS9/RPS4 depicting the central motif that confers nucleolar localization and with the conserved arginine residues mutated to alanine (R109A) and (R127A) shown. Abbreviations: Hs- *Homo Sapiens*, Mm – *Mus Musculus*, Xt- *Xenopus Tropicalis*, Dr – *Danio Rerio*, Dm – *Drosophila Melanogaster*, Ce- *Caenorhabditis Elegans*, Sc – *Saccaromyces Cerevisiae*, Ec- *Escherichia Coli*. B.) The combined mutation of R109 and R127 residues to alanine disrupted the cytoplasmic/nucleolar staining pattern seen in wt RPS9-FLAG expressing cells. A subset transfected cells displayed a more intensely stained sub-nucleolar structure as is indicated with an asterix (*) in the figure, while other transfected cells had such structures in the nucleolar proximity as indicated with the (¤) symbol. Plasmids were transfected transiently into U2OS cells and stained for FLAG expression 24 hours after transfection. Bar 10 µM.

The goal was now set to find important amino acid residues within the RPS9 NoLS with regard to nucleolar localization. In particular arginine residues appear important in many NoLS. Arginine residues within the RPS9 NoLS were indicated by conducting multiple sequence alignments, analysis of crystal structures and literature mining. Previous studies on RPS9 did not resolve exactly of how it binds to RNA [66], [67]. Sequence and crystal structure determination suggested a large RNA binding surface on one side of RPS9. Similarly, RNA-protein interaction studies revealed a complex interplay between different domains in RPS9 and ribosomal or messenger RNA [68]. The central residues R93 and R111 are strictly conserved in RPS4/RPS9 proteins in eukaryotes, prokaryotes and Archaea, and correspond to residues R109 and R127 in human RPS9 [69] (Figure 5A). Davies et al., proposed that R93 and R111 are putative RNA binding residues [66]. These two residues coordinate a sulphate that may mimic a phosphate group on the backbone of a bound RNA [66], [67]. The combined change of R109 and R127 residues to alanine within the full length RPS9^1–194^-FLAG led to an altered cellular localization. The RPS9^1–194(R109A R127A)^-FLAG mutant protein displayed an even nuclear staining without any cytoplasmic staining in the majority (97%) of transfected cells (Figure 5B). While the nucleolar localization was not lost, the pattern was strikingly different in a number of ways compared to wt RPS9-FLAG. First, the mutant protein did not localize throughout the entire nucleolar area as the wt protein did, but was in a subset of cells confined to a smaller region within the nucleolus. Second, not all nucleoli within a cell stained positive for the mutant protein as seen in some transfectants. Third, in a few transfected cells the area with most intense staining was to be found in structures in proximity to the nucleolus. In summary, residues R109 and R127 within the RPS9 NoLS are together critical for the normal localization of RPS9.

### Binding and Co-localization of NPM1 with Different RPS9-FLAG Mutants

**Figure 6:**
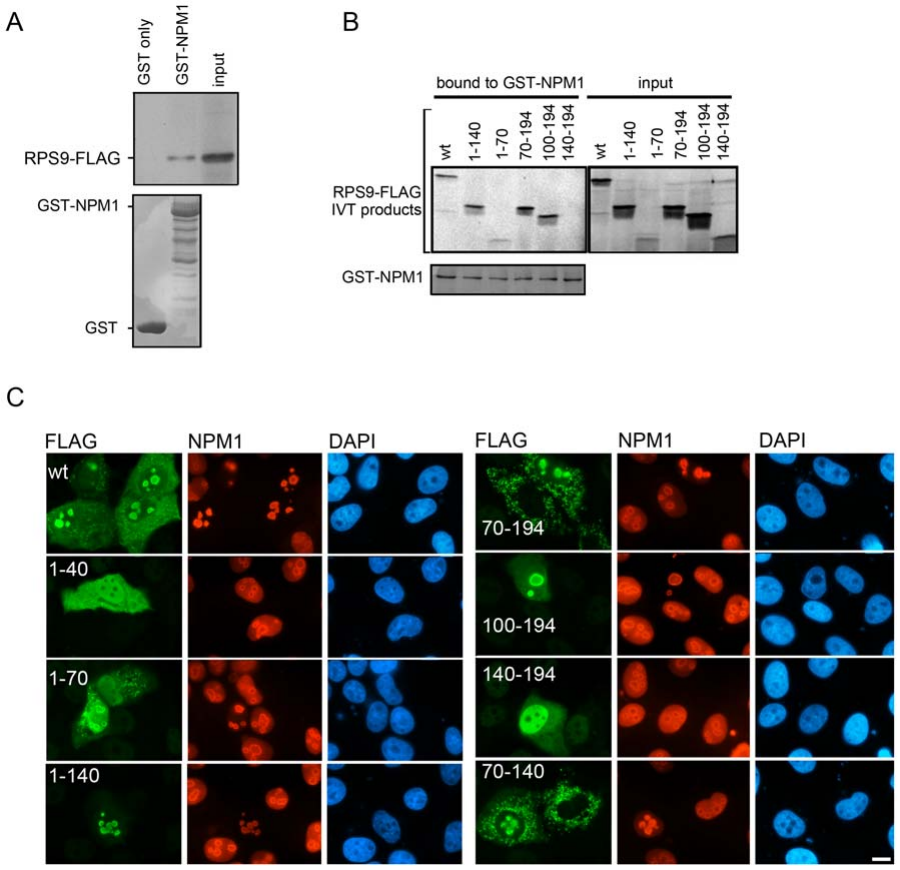
Binding and co-localization of different RPS9 domains with NPM1. A.) In vitro translated RPS9-FLAG binds to GST-NPM1 but not GST. RPS9-FLAG was incubated with GST or GST-NPM1 on beads and bound product was visualized by autoradiography (30% of input is shown). B.) In vitro translated RPS9-FLAG deletion mutants and purified GST-NPM1 revealed that fragments 1–70, 1–140, 70–194, and 100–194 but not RPS9^140–194^-FLAG were able to bind GST-NPM1. The binding of radiolabeled RPS9-FLAG to GST or GST-NPM1 was detected by autoradiography (20% of input is shown). Coomassie brilliant blue staining of the gel demonstrated equivalent levels of input GST. C.) Co-localization of NPM1 and RPS9-FLAG. U2OS cells were transfected with wt RPS9-FLAG and RPS9-FLAG deletion mutants as indicated in the figure. Cells were double stained for FLAG and NPM1 expression using a rabbit polyclonal anti-FLAG antibody and an NPM1 monoclonal antibody. Bar 10 µM.

Enforced expression of NPM1 was previously shown to promote an increased nucleolar abundance of RPS9-FLAG and endogenous RPS9 [42]. This effect could be indirect given that NPM1 has a role in maintaining the nucleolar structure [70]. However, NPM1 can bind to peptides with NLS rich regions of the SV40 LT type and stimulate their nuclear import [35], [36]. Before exploring this issue in the case of RPS9 it was necessary to determine the regions in RPS9 that binds to NPM1. Confirming the interaction between RPS9 and NPM1, it was found that in vitro translated RPS9-FLAG bound to bacterially produced GST-NPM1 (Figure 6A). To identify the NPM1 interaction domain(s) in RPS9, additional in vitro binding assays were performed with purified GST-NPM1 and in vitro translated RPS9-FLAG mutants. This experiment revealed that fragment 1–70 and 70–140, but not 140–194, could bind NPM1, demonstrating that there are at least two binding sites for NPM1 on RPS9 (Figure 6B).

**Figure 7:**
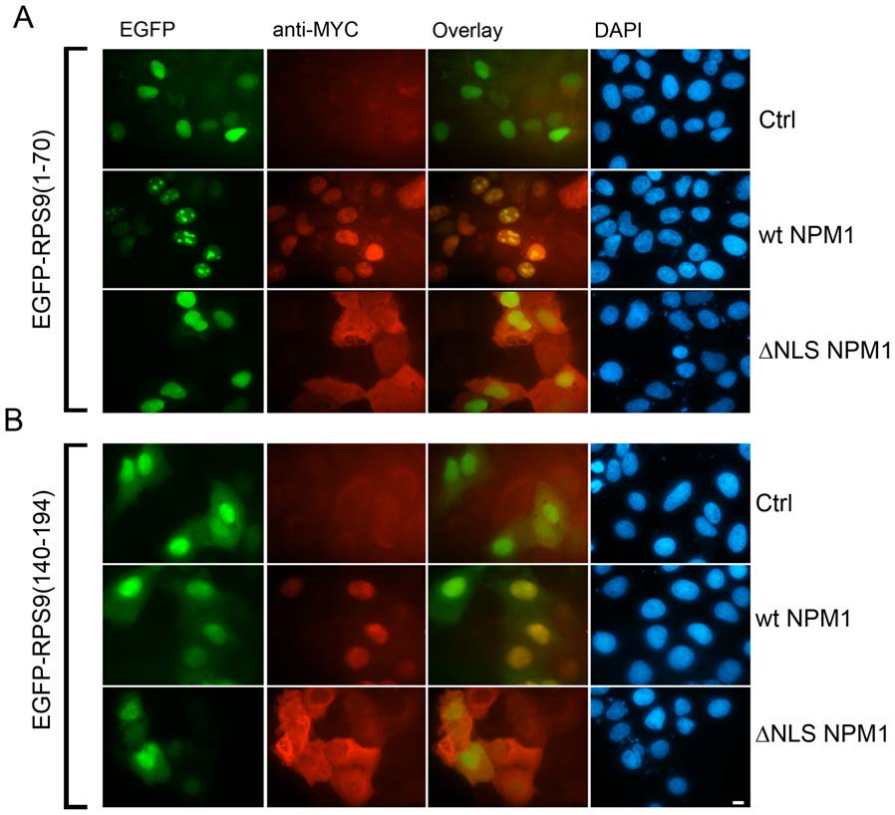
Nucleolar translocation of RPS9 1–70 domain by co-expressed NPM1. A.) Nucleolar localization of EGFP-RPS9^1–70^ was induced by co-expressed Myc-NPM1 (wt), whereas a Myc-NPM1 mutant lacking its nuclear localization signal (Myc-NPM1ΔNLS) failed to do so. Cells were stained for expression of wt and mutant Myc-NPM1. B.) Myc-NPM1 (wt) or Myc-NPM1 mutant did not induce any alteration in the localization of EGFP-RPS9^140–194^. Bar 10 µM. U2OS cells were transfected with plasmids as indicated in the figure and fixed 24 hours after transfection. Cells were stained for Myc expression. Corresponding merged and DAPI images are also shown. Bar 10 µM.

Next, cells expressing various RPS9-FLAG mutants were double stained for FLAG and endogenous NPM1 (Figure 6C). When cells were fixed in formalin, the NPM1 antibody reacted with nucleoplasmic and nucleolar NPM1. In particular this antibody preferentially recognized NPM1 at the rim of the nucleolus, but an identical staining for NPM1 was observed in cells overexpressing the protein with a Myc-tag (Myc-NPM1) (Figure 7). It turned out that in cells expressing RPS9-FLAG, the nucleoplasmic staining of NPM1 was diminished. Instead the nucleolar staining became more distinct. Indeed, all RPS9-FLAG mutants able to bind NPM1 *in vitro* and that localized to nucleoli also altered the endogenous NPM1 staining pattern including RPS9 fragments 1–70, 1–140, 70–194 and 100–194. The altered NPM1 staining may reflect a nucleolar translocation of nucleoplasmic NPM1 by RPS9, reduced total cellular levels of NPM1 or a combination of both. Fragments 1–40 and 70–140 did not translate well and were only evaluated by immunofluorescence.

NPM1 is an abundant protein in tumor cell lines [38] and hence NPM1 is more likely to regulate RPS9 localization than the other way around. To provide additional experimental support for the idea that NPM1 may promote RPS9 nucleolar localization, advantage was taken of the 1–70 fragment of RPS9 that lacks one of the NPM1 binding sites and is predominantly nucleoplasmic. Enforced expression of Myc-NPM1 dramatically altered EGFP-RPS9^1–70^ subcellular distribution directing it to nucleoli in a majority of the cells (87% of co-transfected cells) (Figure 7A). In contrast, a non-nucleolar Myc-NPM1ΔNLS mutant did not induce changes in the localization of EGFP-RPS9^1–70^. As an additional control, EGFP-RPS9^140–194^ that did not bind to NPM1 was also not re-located to nucleoli (Figure 7B). The translocation effect was so pronounced that it is difficult to explain it simply by a general effect on the nucleolar structure induced by NPM1, for instance enlarged nucleoli that would allow for more “storage” of the fusion protein. Indeed, inspection of phase contrast images revealed that the nucleolar structure in cells expressing moderate levels of Myc-NPM1 was not visibly altered (Figure S4).

### RPS9-ΔN Mutant Induces Changes in Nucleolar Structure

**Figure 8:**
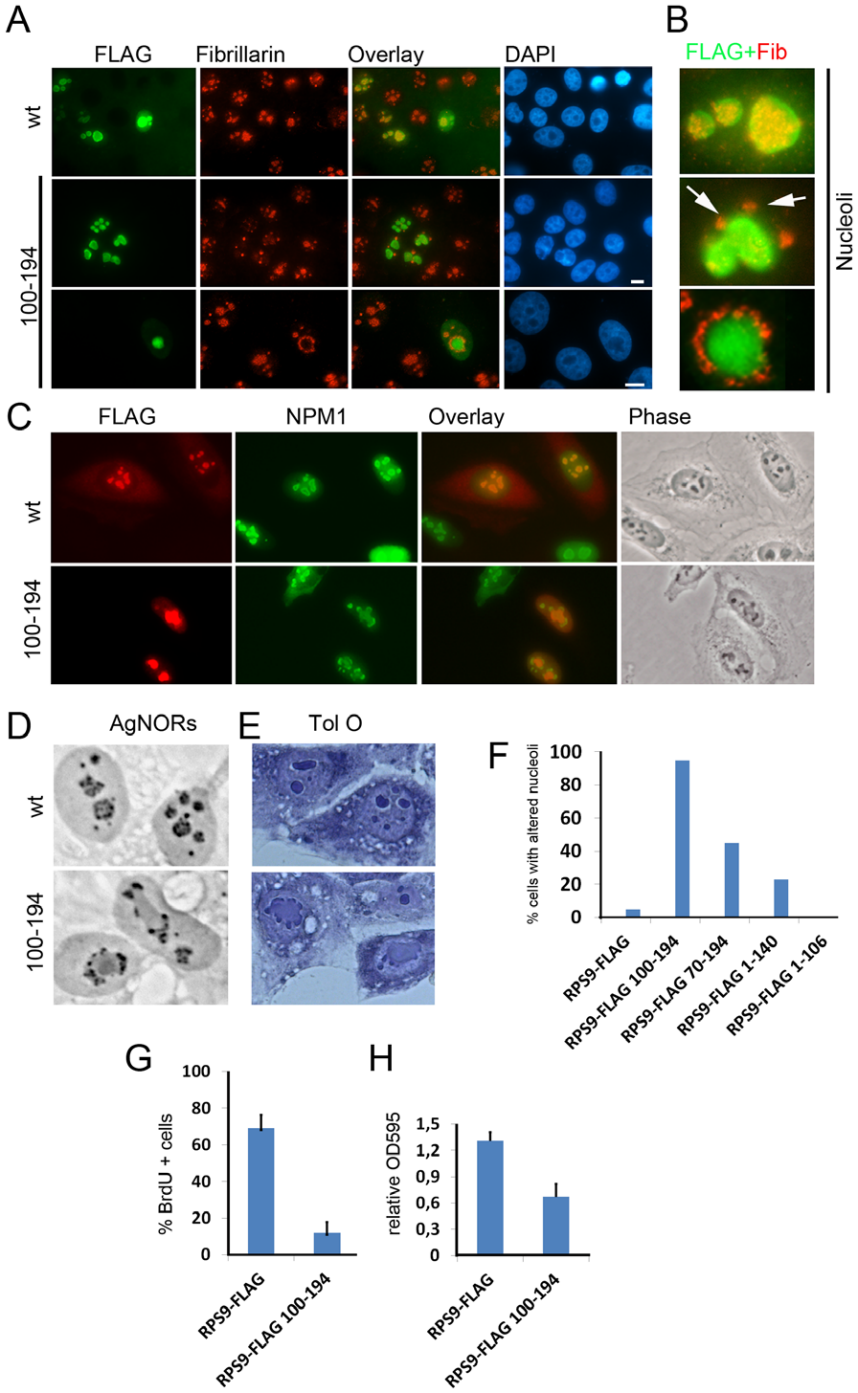
Altered nucleolar morphology in cells expressing RPS9-FLAG mutant. A.) Double staining of wt RPS9-FLAG (green) or RPS9-FLAG^100–194^, and endogenous fibrillarin (red). Nuclei were counterstained with DAPI. B.) Zoom-in images of the nucleolus revealed the distribution of fibrillarin in wt and mutant expressing cells in relation to FLAG. Two different patterns of fibrillarin staining are shown for the RPS9 FLAG mutant expressing cells, namely foci and ring-like. The distinct patterns in mutant expressing cells are indicated with white arrows. Bar 10 µM. C.) Double staining of wt RPS9-FLAG (red), or RPS9-FLAG^100–194^ respectively, and endogenous NPM1 (green) in addition to the corresponding phase contrast images. D.) Silver staining of nucleolar organizer regions (AgNOR) in cells expressing wt or mutant RPS9-FLAG revealed the re-location of silver granules to the surface of a nucleolar expanding mass. Objective 40x. E.) Acidic Toluidine Blue O staining of nucleoli in wt and mutant RPS9-FLAG expressing cells demonstrated that nucleoli had coalesced into one large big nucleolus that was more faintly stained than in wt nucleoli. Objective 40x. F.) Quantification as expressed in percent of transfected cells that displayed altered nucleolar morphology according to DAPI staining and NPM1/fibrillarin localization. G.) Reduced incorporation of BrdU in RPS9-FLAG mutant expressing cells. Data were averaged from at least three independent experiments in which 200 or more cells were counted per sample. Error bars indicate the standard deviation. H.) Bradford measurement (OD595) of the relative protein content in cell lysates from cultures of U2OS cells transfected with wt or mutant RPS9-FLAG. Error bars indicate the standard deviation.

A subset of RPS9 mutants may affect the nucleolar structure or function as indicated by its altered morphology observed by DAPI. Double immunostaining of cells expressing RPS9^100–194^-FLAG revealed that fibrillarin, a nucleolar protein expressed in the nucleolar dense fibrillar centers [57], had become translocated to the surface of an apparently expanding and merging nucleolar area (Figure 8A–B). Fibrillarin was located to either ring-like structures or confined to distinct foci (Figure 8B) in contrast to the more even intra-nucleolar fibrillarin staining seen in untransfected cells or in cells expressing wt RPS9-FLAG. Nucleoli are devoid of dense DNA and they therefore appear as very dark regions when compared with the rest of the nucleus when using DAPI. Staining with DAPI revealed expanding nucleoli in cells expressing RPS9^100–194^-FLAG (Figure 8A). Double immunostaining confirmed that NPM1 at least partially co-localized with RPS9^100–194^-FLAG and the altered nucleolar morphology could be seen by light microscopy as well (Figure 8C).

To understand the change in nucleolar morphology in greater detail additional stainings of the nucleolus were made. The nucleolar organizer regions (NORs) are composed of hundreds of repeated copies of rDNA genes. NORs include regions that are highly condensed and regions that are de-condensed, the latter corresponding to regions at which associated proteins stain intensively with silver (Ag-NORs) and where active rRNA gene transcription is thought to occur [71]. Analysis of Ag-NOR proteins stained with silver nitrate in U2OS cells expressing RPS9^100–194^-FLAG revealed that the Ag-NOR granules were somewhat smaller in size and instead distributed on the surface of a less dense but still distinct nucleolar mass similar to fibrillarin (Figure 8D). Next, unfixed U2OS cells transfected with wt or mutant RPS9 were stained with acidic Toluidine Blue O stain. At acidic pH this dye stains RNA and results in prominent nucleolar staining due to the high content of ribosomal RNA [57]. This staining confirmed the grossly expanding nucleolar mass as was indicated by DAPI staining above (Figure 8E). Importantly, the nucleolar enlargement and re-structuring was detected in cells expressing RPS9-FLAG^70–194^ and RPS9-FLAG^1–140^ albeit at a lower frequency (Figure 8F), but was not observed in cells transfected with wild type RPS9-FLAG. In cells expressing RPS9-EGFP^106–194^, the effect on the nucleolus was less pronounced and it took until day two after transfection for this phenotype to become evident in ∼30% of the cells (not shown), presumably EGFP has a shielding effect. The impact on cell proliferation in cultures transfected with the mutant construct was severe. Cells expressing RPS9^100–194^-FLAG had a dramatically reduced incorporation of BrdU (12±6% versus wt RPS9-FLAG 69±7%, p<0.01) (Figure 8G). Moreover, cell cultures transfected with RPS9^100–194^-FLAG construct had lower total protein content (Figure 8H), and did not produce any colonies or stable cell lines upon selection. These findings taken together demonstrate that the expression of RPS9^100–194^-FLAG mutant inhibited cell proliferation.

## Discussion

Ribosome biogenesis is thought to require highly efficient nuclear import of ribosomal proteins [5]. The sequence of RPS9 is predicted to contain multiple NLSs and by an experimental approach undertaken here RPS9 was found to have three regions capable of inducing a predominant nuclear accumulation of EGFP. The first region is located in the N-terminus (aa 1–70) that is predicted to contain two overlapping bi-partite NLSs. The second region is located in the C-terminus of RPS9, indeed predicted to have a mono-partite NLS (aa 140–194). The third region competent for nuclear localization was found within the central parts of the protein (aa 106–140). Several other RPs have been found to have multiple NLSs including RPS25, RPL5, RPS6, and RPL7A [28], [31], [64], [72], [73]. Many of these NLSs consist of basic amino acid stretches, which show similarities to classical NLSs however, others do not fit into the consensus sequences. It is not clear why some RPs have many NLSs. One prevailing idea is that it serves to provide for the rapid nuclear import required and that the many NLSs may reflect a redundancy of nuclear import pathways thus representing binding sites for different transport factors. The exact structure of the NLSs in RPS9 remains to be deduced by a comprehensive site directed mutagenesis approach.

Following nuclear import, the majority of RPs must travel to the nucleolus in order to be able to participate in ribosome subunit assembly. Nucleolar localization could occur by diffusion through the nucleoplasm and retention by interaction with other nucleolar components or it could be a more active process that relies on specific nucleolar shuttling proteins. RPS9 localizes to nucleoli similar to other RPs [42], [53]. A central region (aa 106–140) was found here to be important for the nucleolar localization of RPS9. When the RPS9 NoLS region was fused with the regions encompassing NLS-1 and NLS-2, the nucleolar localization appeared more intense as was seen with fragment 1–140 and 106–194. Therefore, both the NLS-1 and the NLS-2 containing region may function to facilitate the nucleolar localization of RPS9. That a potent NoLS is residing in the central region of RPS9 did come as a surprise. Overall, it appears that prediction of NLS and NoLS in RPs still remains a challenge and in need of experimental validation.

NoLSs in RPS6, RPS25 and RPS7 reside in evolutionary highly conserved sequences [64]. The results obtained with RPS9 suggest that it too, belongs to this category of RPs. That the NoLSs in a subset of RPs reside in peptide clusters conserved over millions of years and across species [64], implies that these motifs evolved to bind rRNA (or other important structure in the ribosome), rather than to become a genuine signal for nucleolar localization. It is possible that rRNA binding by RPS9 and its nucleolar localization could be determined by the same residues. RPS9 binding to rRNA binding might also be of importance for the RPS9-NPM1 association. However, it was shown previously that RNase treatment did not reduce the RPS9-NPM1 interaction in vitro [42]. One hypothesis is that NPM1 preferentially binds to RPS9 when it is not bound to rRNA in order to prevent aggregation of RPS9.

The N-terminus of RPS9 (aa 1–70) interacted with NPM1 in vitro, conferred nuclear localization and had some affinity for the nucleolus. Interestingly, according to NoD this N-terminal domain in RPS9 has a rather high probability of nucleolar localization. However, this domain appeared rather weak in its nucleolar targeting activity and I therefore do not consider it to be a genuine NoLS. Nonetheless, the predominantly nucleoplasmic RPS9 mutant 1–70 was more efficiently targeted to the nucleolus by co-expression of NPM1. Conceivably, NPM1 could play a role in promoting the nucleolar localization of RPS9, reminiscent of NPM1 mediated nucleolar targeting of viral proteins and the p14ARF tumor suppressor [35], [36], [74]. Whether NPM1 actively transport proteins with a NoLS, or if NPM1 only plays a role in the retention of them in the nucleolus through the NoLS, remains to be determined. It should also be pointed out that NPM1 is unlikely to be essential for the nucleolar localization of RPS9 given that expression of NPM1 is restricted to animals and has not been found in yeast [75] whereas RPS9 is expressed in all phyla including yeast.

There are a number of pitfalls that one has to keep in mind when evaluating RPS9 mutants. RPS9 is a structurally complex protein and with such an extended RNA binding surface, manipulation of its structure by sequential deletions is likely to have major impact on its folding, rRNA binding and association with NPM1. Therefore, the behaviour of individual RPS9 mutants might therefore not always accurately describe the situation in the context of the wt protein. Moreover, several of the RPS9 mutants displayed localization patterns (mitochondria, nucleolar aggregates) or had a fairly variable localization pattern not observed with the endogenous protein.

The increasing interest concerning RPs and their role in cell growth and proliferation through the RP-MDM2-p53 pathway [76], [77], and also their causative association with human disease syndromes including Diamond Blackfan anemia [78], means that there is a need for a better understanding of RPs. While RPS9 has so far not been associated with Diamond Blackfan anemia, studies on RPS9 and artificial RPS9 mutants may provide clues to the functions and behaviors of other RPs. During the course of this work it was discovered that a subset of RPS9 mutants had a dramatic effect on the nucleolar structure. As was studied in greater detail by using the RPS9^100–194^-FLAG construct, overexpression of the mutant resulted in nucleolar enlargement and altered nucleolar organization. A key component of the nucleolar dense fibrillar center, fibrillarin, was dispersed in a few small spots or ring like structures surrounding the enlarged nucleoli. These spots were also intensely stained with silver. Intriguingly, NPM1 appeared trapped in these enlarged nucleoli. It is possible that the expanding nucleolar area contains an aggregate of the mutant protein mixed with other nucleolar proteins and RNA resulting in structural changes [79].

Expression of the RPS9-FLAG mutant severely impaired cell proliferation as shown by a reduced BrdU incorporation, lowered amounts of total protein, and by the failure to establish stable cell lines. Truncated versions of RPS9 may disrupt ribosome biogenesis by acting as dominant negative mutants. By interfering with rRNA synthesis or pre-ribosome export the mutant may induce nucleolar stress [80]. However, the RPS9^100–194^-FLAG mutant, as well as fragments 1–140 and 70–194 tagged with FLAG, associated with NPM1 and an alternative explanation is that RPS9 mutants directly inhibit NPM1, similar to what has been described for a subset of NPM1 binding peptides [81]–[84]. Inhibition of NPM1 could in turn cause the alterations in nucleolar morphology keeping in mind NPM1’s critical role in the nucleolus [70]. In any case, exactly how RPS9 mutant impair cell proliferation remains to be determined. Future research should also focus on the role of post-translational modifications of RPS9 and if such modifications affect its nucleolar localization, function in ribosome biogenesis, and role in protein synthesis.

## Supporting Information

Figure S1 **RPS9 multiple sequence alignment.** RPS9 sequences from different organisms were aligned using Clustal W and the NLS-1 and NLS-2 motifs as predicted by PSORT II are indicated in green. The NoLS containing domain is indicated in purple. Abbreviations: Hs- *Homo Sapiens*, Rn – *Rattus Norvegicus*, Mm – *Mus Musculus*, Xt- *Xenopus Tropicalis*, Dr – *Danio Rerio*, Bt – *Bos Taurus*, Dm – *Drosophila Melanogaster*, Ce- *Caenorhabditis Elegans*, Sp –. *Schizosaccharomyces Pombe,* Nc *– Neurospora Crassa.*
(TIF)

Figure S2 **Prediction of NoLS and NES in RPS9.** A.) Diagram showing the NoLS prediction score per residue in human RPS9 using NoD. B.) Schematic representation showing nuclear export sequence probability per residue using NetNES.
(TIF)

Figure S3 **Partial mitochondrial localization of RPS9-FLAG deletion mutant.** U2OS cells were transfected with wt RPS9-FLAG or RPS9-FLAG^70–140^ deletion mutants. Cells were incubated with Mitotracker Red, fixed, and stained for FLAG. Merged images show co-localization of the FLAG and Mitotracker Red signals in the case of the mutant protein but not in wt expressing cells. Bar 10µM. Cell nuclei were stained by DAPI.
(TIF)

Figure S4 **Nucleolar translocation of RPS9^1–70^-FLAG induced by co-expression of NPM1.** A.) Nucleolar localization of RPS9^1–70^-FLAG was induced by co-expressed Myc-NPM1 (wt), whereas a cytoplasmic NPM1 mutant did not. Cells were stained for expression of wt and mutant Myc-NPM1 using a rabbit anti-Myc polyclonal antibody and a monoclonal FLAG antibody (M2). Corresponding phase contrast images are shown.
(TIF)

Table S1 **Prediction of nuclear (NLS) and nucleolar localization signals (NoLS) in small subunit ribosomal proteins.** An overall NLS score according to PSORT II is given for each protein, as is the number of predicted NLS, percentage of basic amino acid residues, and the number of predicted NoLS. Note that for some ribosomal proteins the repetitive nature of basic amino acid residues results in a high number of NLS in the case of e g RPS6, RPS8, RPS27A and RPS27.
(PDF)
