# Supplementary figures and images for: Elucidation of Motifs in Ribosomal Protein S9 That Mediate Its Nucleolar Localization and Binding to NPM1/Nucleophosmin
Source: PLoS One. 2012 Dec 20;7(12):e52476. doi: 10.1371/journal.pone.0052476 (PMC3527548; doi:10.1371/journal.pone.0052476)

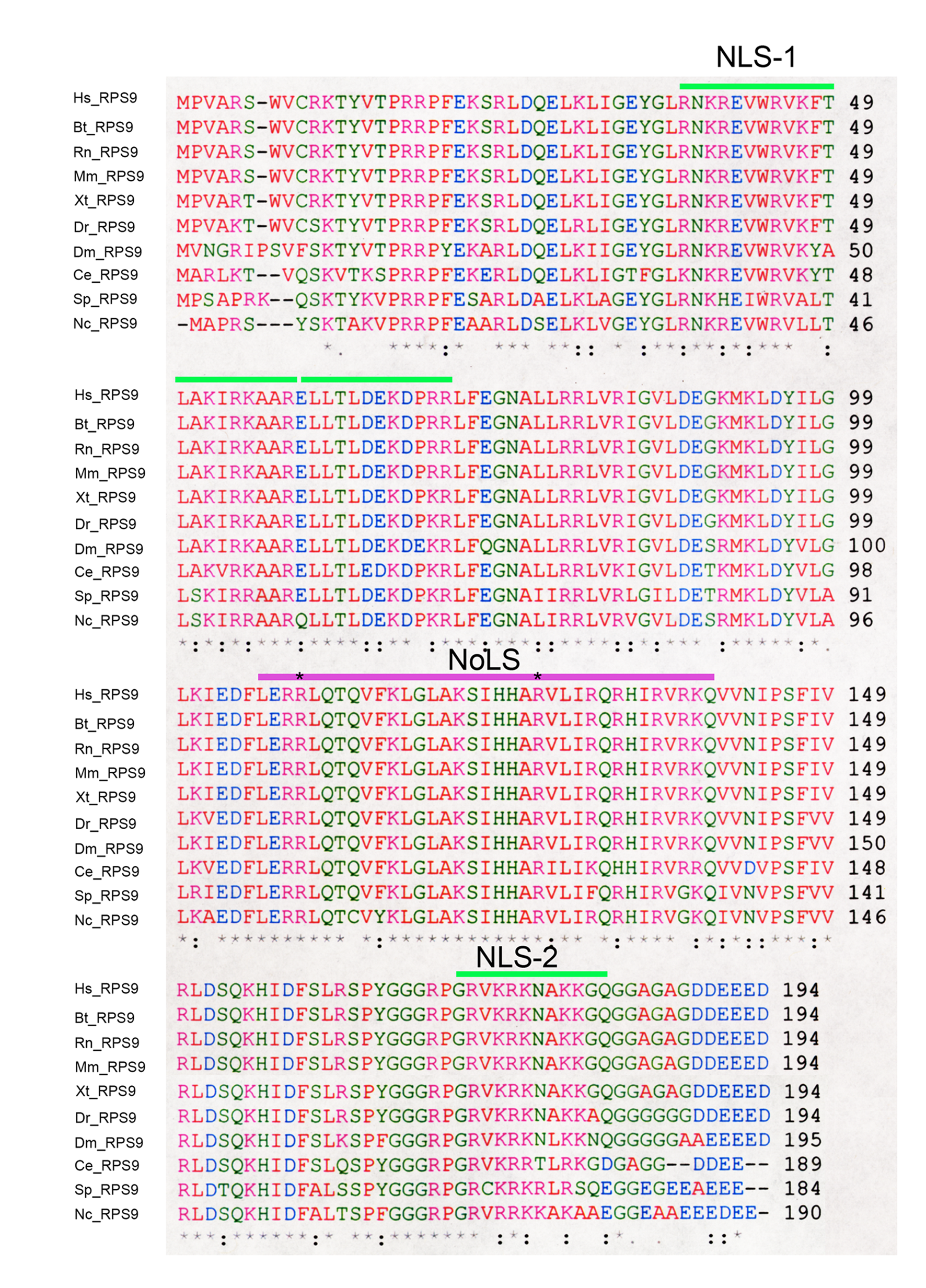

Supplement: Figure S1 — RPS9 multiple sequence alignment. RPS9 sequences from different organisms were aligned using Clustal W and the NLS-1 and NLS-2 motifs as predicted by PSORT II are indicated in green. The NoLS containing domain is indicated in purple. Abbreviations: Hs- Homo Sapiens, Rn – Rattus Norvegicus, Mm – Mus Musculus, Xt- Xenopus Tropicalis, Dr – Danio Rerio, Bt – Bos Taurus, Dm – Drosophila Melanogaster, Ce- Caenorhabditis Elegans, Sp –. Schizosaccharomyces Pombe, Nc – Neurospora Crassa. (TIF) [file pone.0052476.s001.tif]

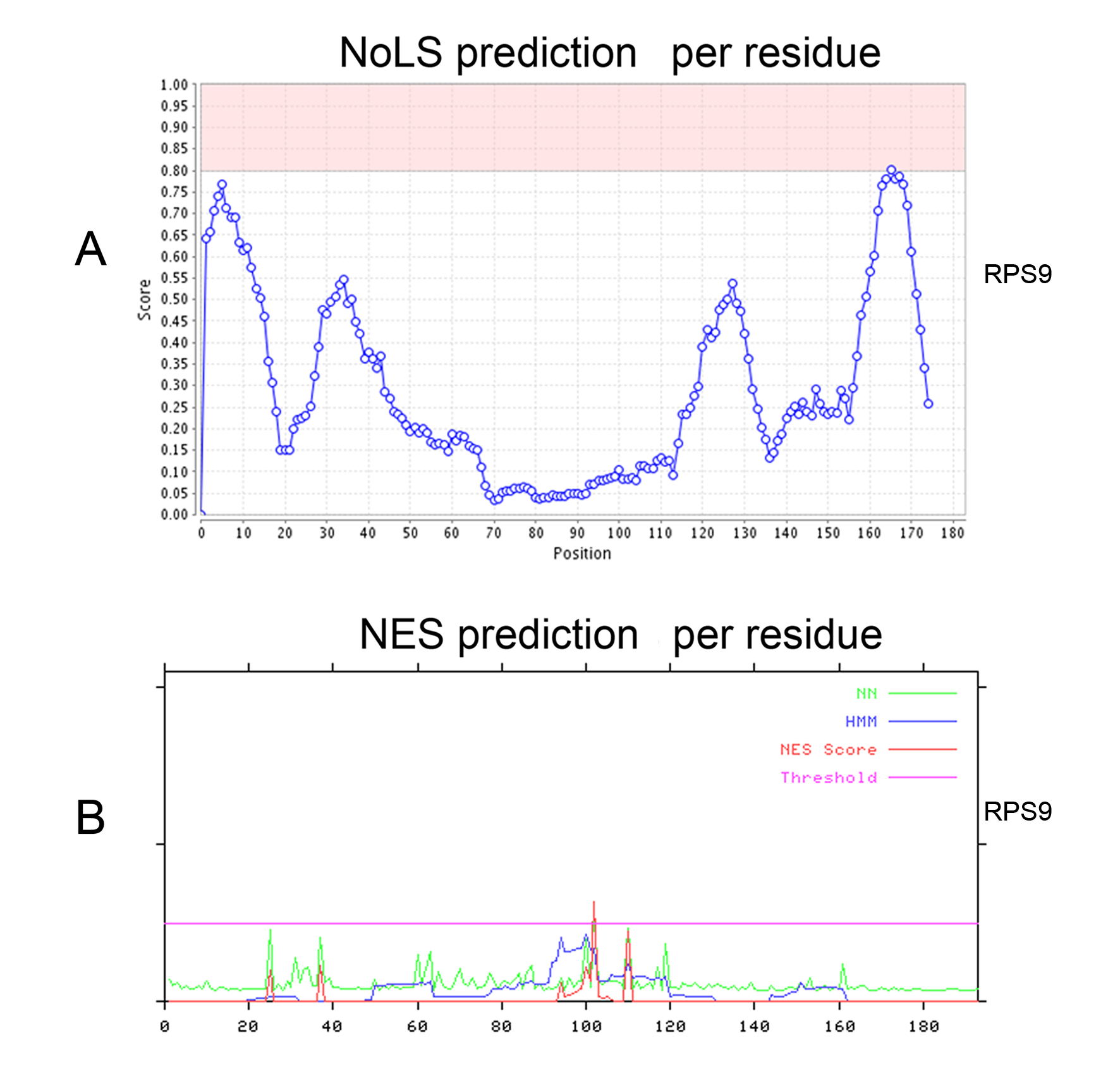

Supplement: Figure S2 — Prediction of NoLS and NES in RPS9. A.) Diagram showing the NoLS prediction score per residue in human RPS9 using NoD. B.) Schematic representation showing nuclear export sequence probability per residue using NetNES. (TIF) [file pone.0052476.s002.tif]

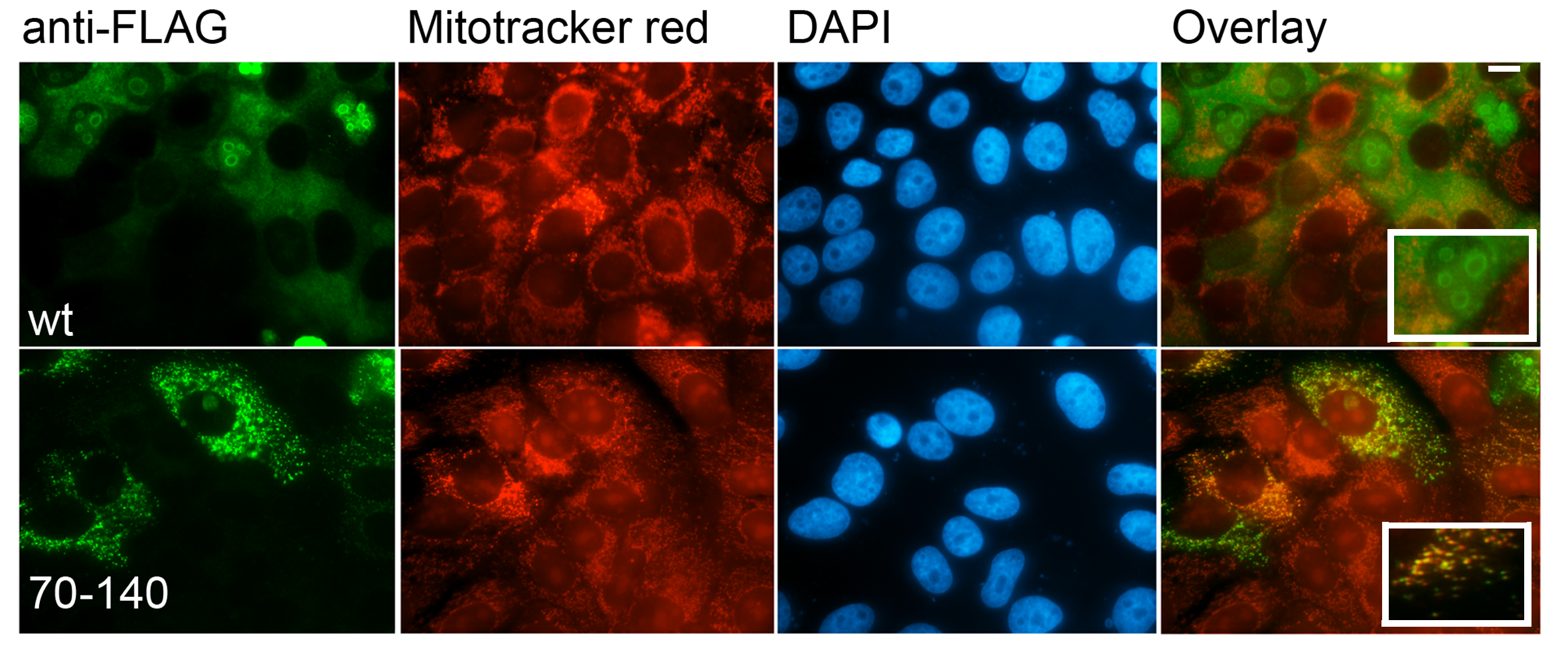

Supplement: Figure S3 — Partial mitochondrial localization of RPS9-FLAG deletion mutant. U2OS cells were transfected with wt RPS9-FLAG or RPS9-FLAG70–140 deletion mutants. Cells were incubated with Mitotracker Red, fixed, and stained for FLAG. Merged images show co-localization of the FLAG and Mitotracker Red signals in the case of the mutant protein but not in wt expressing cells. Bar 10µM. Cell nuclei were stained by DAPI. (TIF) [file pone.0052476.s003.tif]

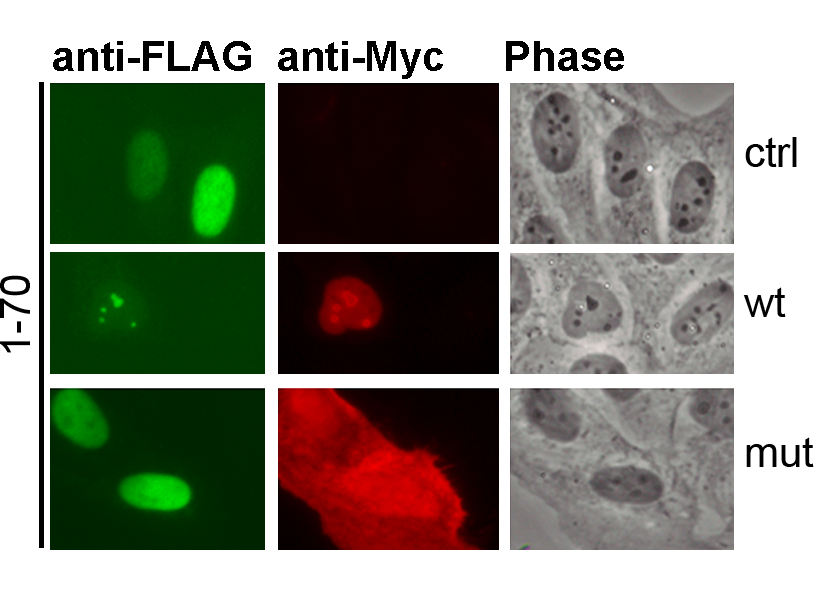

Supplement: Figure S4 — Nucleolar translocation of RPS91–70-FLAG induced by co-expression of NPM1. A.) Nucleolar localization of RPS91–70-FLAG was induced by co-expressed Myc-NPM1 (wt), whereas a cytoplasmic NPM1 mutant did not. Cells were stained for expression of wt and mutant Myc-NPM1 using a rabbit anti-Myc polyclonal antibody and a monoclonal FLAG antibody (M2). Corresponding phase contrast images are shown. (TIF) [file pone.0052476.s004.tif]
