# Supplementary material for: Elucidation of Motifs in Ribosomal Protein S9 That Mediate Its Nucleolar Localization and Binding to NPM1/Nucleophosmin
Source: PLoS One. 2012 Dec 20;7(12):e52476. doi: 10.1371/journal.pone.0052476 (PMC3527548; doi:10.1371/journal.pone.0052476)
Supplement: Table S1 — Prediction of nuclear (NLS) and nucleolar localization signals (NoLS) in small subunit ribosomal proteins. An overall NLS score according to PSORT II is given for each protein, as is the number of predicted NLS, percentage of basic amino acid residues, and the number of predicted NoLS. Note that for some ribosomal proteins the repetitive nature of basic amino acid residues results in a high number of NLS in the case of e g RPS6, RPS8, RPS27A and RPS27. (PDF) [file pone.0052476.s005.pdf]

Supplementary Table S1. **Prediction of nuclear (NLS) and nucleolar localization signals (NoLS) in small subunit ribosomal proteins. The percentage % of basic residues is also indicated. Prediction of NLS was made with PSORT II and prediction of NoLS using NoD.**

RP	NLS score	NLS	basic residues (%)	NoLS
RPS2	-0,47	0	16,4	0
RPS3	-0,16	1	15,6	0
RPS3A	0,27	2	19,7	1
RPS4X	-0,47	0	18,6	0
RPS4Y	-0,47	0	18,6	0
RPS5	-0,47	0	16,2	0
RPS6	2,6	7	27,3	2
RPS7	0,12	2	19,6	1
RPS8	5,79	13	26,6	1
RPS9	1,81	3	23,7	1
RPS10	-0,04	1	18,8	0
RPS11	1,16	2	22,2	0
RPS12	-0,22	1	15,9	0
RPS13	0,97	1	21,2	0
RPS14	-0,04	1	17,9	0
RPS15	1,28	2	21,4	2
RPS15A	-0,29	1	16,9	0
RPS16	0,48	0	20,5	0
RPS17	0,36	2	19,3	0
RPS18	0,48	0	23,7	0
RPS19	-0,47	0	18,6	0
RPS20	-0,47	0	19,3	0
RPS21	-0,47	0	14,8	0
RPS23	0,48	0	22,4	1
RPS24	1,71	2	27,1	1
RPS25	2,02	4	26,4	1
RPS26	0,79	1	25,2	2
RPS27	0,85	6	17,9	1
RPS27A	5,5	12	22,4	1
RPS28	0,48	0	20,3	0
RPS29	-0,47	0	19,6	0
RPS30	0,41	3	17,3	1
